# Second-generation trabecular micro-bypass stent implantation: Retrospective analysis after 12- and 24-month follow-up

**DOI:** 10.1186/s40662-019-0169-7

**Published:** 2020-01-10

**Authors:** Raphael Neuhann, Tobias Neuhann

**Affiliations:** Ophthalmologikum Dr.Neuhann, Augentagesklinik Marienplatz, Marienplatz 18-19, 80331 Munich, Germany

**Keywords:** Glaucoma, MIGS, iStent inject, Trabecular, Safety, Stent, Bypass

## Abstract

**Background:**

The study aimed to investigate the 24-month safety and efficacy of implantation of two second-generation iStent inject trabecular micro-bypass stents with concomitant cataract surgery.

**Methods:**

This consecutive case series included 164 eyes of 109 patients implanted with the iStent inject® device with concomitant cataract surgery. The series was comprised of eyes with primary open-angle glaucoma (*n* = 84), pseudoexfoliation glaucoma (*n* = 42), normal-tension glaucoma (*n* = 18), and ocular hypertension (*n* = 20). All 164 eyes reached 9–14 months of follow-up (“12-month consistent cohort”), with a subset of 88 eyes reaching 21–26 months of follow-up (“24-month consistent cohort”). Performance outcome measures included intraocular pressure (IOP) and number of glaucoma medications. Safety outcomes included intra- or postoperative complications, the need for secondary procedures and corrected distance visual acuity. Comparisons of change in continuous (e.g., IOP) and categorical (e.g., proportions of eyes on zero medications) measures between baseline and postoperative times were made with the paired t-test and McNemar’s chi-squared test, respectively.

**Results:**

At 12 months postoperatively, IOP was reduced by 25.5% (from 20.0 ± 5.5 mmHg to 14.9 ± 2.0 mmHg; *p* < 0.001); at 24 months postoperatively, IOP was reduced by 26.6% (from 20.3 ± 6.1 mmHg to 14.9 ± 1.9 mmHg; *p* < 0.001). At 12 months postoperatively, mean number of glaucoma medications was reduced by 85.0% (from 2.0 ± 1.0 to 0.3 ± 0.8 medications; *p* < 0.001); at 24 months postoperatively, mean number of medications was reduced by 81.0% (from 2.1 ± 1.1 to 0.4 ± 0.8 medications; *p* < 0.001). After 12 months, 96.3% of eyes had an IOP ≤ 18 mmHg and 58.5% of eyes had an IOP ≤ 15 mmHg, with 81.1% of eyes free of any medication, compared to 1.8% of eyes medication-free in the 12-month cohort at baseline. After 24 months, 98.9% of eyes had an IOP ≤ 18 mmHg and 53.4% of eyes had an IOP ≤ 15 mmHg, with 72.7% free of medication compared to 1.1% of eyes medication-free in the 24-month cohort at baseline. Overall, a high safety profile was observed with no significant postoperative complications.

**Conclusions:**

The insertion of iStent inject (comprised of two second-generation trabecular micro-bypass stents) with cataract surgery effectively provides a sustained reduction in IOP with a markedly improved medication burden up to 24 months postoperatively.

## Background

Due to demographic development and increasing life expectancy, glaucoma is expected to increase to 80 million people by 2020 [1]. Glaucoma is one of the major causes of irreversible blindness worldwide. Intraocular pressure (IOP) is the most important risk factor for glaucoma development, along with other influencing factors such as age, family history, previous eye injuries, operations or inflammation. Therefore, the maintenance of visual acuity and visual function to preserve the quality of life is the primary goal of glaucoma treatment. All therapeutic interventions, whether pharmaceutical or surgical, are aimed at decreasing IOP [2]. One of the most common treatment options for most forms of glaucoma is the use of topical medication, in the form of eye drops. Consistent patient adherence to therapy recommendation is of great importance. Due to a lack of compliance that can range from approximately 30 to 80% [3], many patients do not use eye drops in a medically prescribed manner - or worse, do not use their drops at all [4, 5]. Physical disabilities such as tremor or arthritis can also affect the administration of eye drops leading to inappropriate medication usage. Current glaucoma procedures such as selective laser trabeculoplasty, trabeculectomy, and implantation of glaucoma drainage devices as an alternative to drug therapy are often associated with complications [6–8] or when successful do not have enduring efficacy [9].

The surgical treatment of open-angle glaucoma has undergone tremendous innovation in the past decade with the emergence of minimally invasive glaucoma surgery (MIGS), a growing space of minimally invasive procedures that aim to safely and effectively lower IOP and reduce dependency on medication use. The first-generation iStent*®* and second-generation iStent inject® (Glaukos, San Clemente, CA, USA) are trabecular micro-bypass MIGS devices implanted ab internally through the same cataract incision when performed in conjunction with cataract surgery. The stents target the conventional aqueous humor outflow pathway enhancing egress via the trabecular meshwork, the site of greatest outflow resistance. The stents (one stent per iStent device, two stents per iStent inject device) are inserted into the trabecular meshwork to create a bypass allowing the aqueous humor to flow directly from the anterior chamber into Schlemm’s canal. The physiological preservation of the trabecular meshwork ensures a natural episcleral counterpressure of 8–11 mmHg with minimal risk of hypotension. The increase of outflow through the inferonasal quadrants has a significant influence on the IOP reduction due to the high number of collector channels known to exist in that region. Studies have shown both iStent and iStent inject offer significant reductions in IOP and drug exposure with a relatively low long-term risk [10].

The iStent and iStent inject devices have been shown to considerably, sustainably, and safely reduce IOP and medications in patients diagnosed with primary open-angle glaucoma, pseudoexfoliation glaucoma, ocular hypertension, normal-tension glaucoma, or pigmentary glaucoma, and either with or without cataract surgery [11–42]. This case series presents the results after implantation of the iStent inject with concomitant cataract surgery 24 months postoperatively in a routine clinical setting in Germany, with one of the largest and longest experiences with the devices.

## Methods

### Study design

Consecutive patients undergoing iStent inject implantation in combination with cataract surgery were included in this case series. Data were collected at 1 day, 1 week, 1 month, 2–4 months, 4–6 months, 9–14 months, and 21–26 months after surgery. In reporting study time points in this manuscript, the terms “9–14 months” and “12 months” are used interchangeably; and the terms “21–26 months” and “24 months” are used interchangeably. All procedures were performed by a single surgeon (TN) at a single site. Due to the retrospective analysis of anonymized data, this study did not need to undergo ethics approval. The handling of the data followed the data protection directives and the Declaration of Helsinki.

### Device description

iStent inject, the second-generation trabecular micro-bypass stent system, encompasses two biocompatible, medical-grade, nickel-free trabecular micro-bypass stents that are pre-loaded on a single injector to permit bypass of two separate areas of the trabecular meshwork in a single procedure. In comparison to the first-generation iStent device, the iStent inject stents are smaller in size (0.36 mm × 0.23 mm) and each has 4 lateral outlet lumina (rather than one in the iStent), thereby facilitating multidirectional flow and increasing access to more collector channels. Each stent can assess up to 6 clock-hours of aqueous outflow and is designed to carry 2.5 μL/min of aqueous humor, the maximum amount typically produced by the human body. In addition, the stents have a uniform shape (rather than right/left orientation) and are implanted perpendicularly directly into Schlemm’s canal, increasing procedural efficiency and easing the learning curve helping the physician incorporate the device into his/her surgical armamentarium.

### Main outcome measures

Primary outcome measures included mean postoperative IOP via Goldmann applanation tonometry and ocular hypotensive medication use. The consistent cohort data for both 12 months and 24 months are reported. Proportional analyses were completed for patients with an IOP ≤ 15 mmHg and an IOP ≤ 18 mmHg, in addition to ≥ 20% IOP decrease from baseline at the follow-up visits. Medication use was analyzed and computed to note changes in medication burden from baseline. Visual acuity outcomes were also evaluated. Results were stratified for the type of glaucoma.

In patients who contributed information on both eyes to the study, each eye was considered an independent unit of observation for the purposes of the study. A Pearson correlation coefficient was calculated to provide a measure of the correlation between eyes in postoperative IOP reduction at 12 months. To summarize the pre- and postoperative data, descriptive statistics were used. Preoperative IOP was compared to 12-month and 24-month IOP using the t-test for dependent samples. The McNemar test was used to compare pre- and postoperative proportions of eyes on zero medications or eyes on ≥ 3 medications. A *p*-value of 0.05 or less was considered statistically significant.

## Results

### Baseline parameters

A total of 164 eyes of 109 patients with primary open angle glaucoma (POAG, 84 eyes), pseudoexfoliation glaucoma (PEX, 42 eyes), normal tension glaucoma (NTG, 18 eyes), and ocular hypertension (OH, 20 eyes) completed the 12-month follow-up and were included in this case series. The mean age for the total cohort was 76.7 years with a slight female predominance. Table 1 shows the preoperative demographic and ocular data. Of these eyes, 88 eyes (46 with POAG, 19 with PEX, 10 with NTG and 13 with OH) completed the 24-month follow-up. The data for the consistent cohort of eyes at both 12 and 24 months were analyzed.

**Table 1:** Baseline Demographic and Ocular Characteristics

	Total	POAG	PEX	NTG	OH
Number					
patients	109	54	28	12	15
eyes	164	84	42	18	20
Age (years)					
Mean ± SD	76.7 ± 6.8	76.6 ± 7.5	77.3 ± 4.7	79.3 ± 6.5	73.6 ± 7.6
Sex					
male	48	27	11	6	4
female	61	27	17	6	11
Eye					
OD	85	42	21	11	11
OS	79	42	21	7	9
History of glaucoma surgery (# of eyes)	8	4	3	0	1
IOP (12-month cohort)					
N	164	84	42	18	20
Mean (mmHg)	20.0	20.2	20.3	17.1	21.3
SD	5.5	5.6	4.9	5.4	5.6
IOP (24-month cohort)					
N	88	46	19	10	13
Mean (mmHg)	20.3	20.3	21.7	17.3	20.7
SD	6.1	6.1	6.1	5.5	6.2
Medications (12-month cohort)					
N	164	84	42	18	20
Mean	2.0	2.1	2.1	1.7	1.5
SD	1.0	1.0	1.0	0.9	1.0
Medications (24-month cohort)					
N	88	46	19	10	13
Mean	2.05	2.13	2.21	1.90	1.62
SD	1.09	1.13	1.18	0.74	1.04
Eyes on					
0 med	3 (1.8%)	0 (0%)	0 (0%)	0 (0%)	3 (15.0%)
1–2 med	108 (65.8%)	55 (65.5%)	25 (59.5%)	15 (83.3%)	13 (65.0%)
≥ 3 med	53 (32.4%)	29 (34.5%)	17 (40.5%)	3 (16.7%)	4 (20.0%)
N (%)					
CDVA (N)	164	84	42	18	20
Mean LogMAR	0.39	0.41	0.36	0.43	0.35
SD	0.17	0.18	0.14	0.17	0.17
C/D ratio					
Mean	0.55	0.61	0.54	0.55	0.30
SD	0.24	0.21	0.25	0.24	0.20

POAG = primary open angle glaucoma; PEX = pseudoexfoliation glaucoma; NTG = normal tension glaucoma; OH = ocular hypertension; IOP = intraocular pressure; CDVA = corrected distance visual acuity; SD = standard deviation

### IOP evaluation

**Fig. 1:**
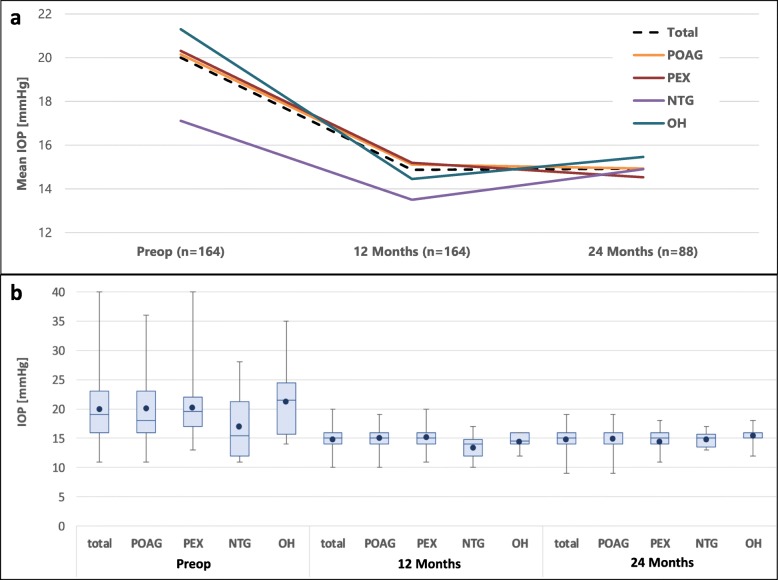
Intraocular pressure (IOP) through 12 and 24 months of follow-up. (a) Mean values presented. (b) Box and whisker plot: box represents interquartile range (25th to 75th percentile); dot represents mean; horizontal line represents median (50th percentile); upper and lower vertical lines represent 95th and 5th percentiles, respectively. POAG = primary open angle glaucoma; PEX = pseudoexfoliation glaucoma; NTG = normal tension glaucoma; OH = ocular hypertension

In the overall cohort, mean IOP was reduced by 25.5% from 20.0 ± 5.5 (mean ± standard deviation) mmHg preoperatively to 14.9 ± 2.0 mmHg at 12 months postoperatively (*p* < 0.001). At the 24-month follow-up, mean IOP was reduced by 26.6% from 20.3 ± 6.1 mmHg preoperatively to 14.9 ± 1.9 mmHg at 24 months (p < 0.001). No significant correlation was found in the IOP reduction between the right and left eyes in bilaterally treated patients at 12 months (*n* = 110 eyes of 55 patients; Pearson correlation coefficient = 0.022, with significance threshold of 0.412). Figure 1 shows the IOP development over the entire follow-up period, grouped by glaucoma type. Statistically significant reductions of IOP at 12 months versus baseline were observed for patients with all glaucoma types (POAG, PEX and OH: *p* < 0.001; NTG: *p* = 0.01). After 24 months, significant IOP reductions vs baseline were found for POAG, PEX and OH (*p* < 0.001 for POAG and PEX; *p* = 0.011 for OH). An IOP reduction was also found for NTG (*n* = 10 at 24 months), but this was not statistically significant (*p* = 0.132). Table 2 shows the absolute and percent IOP reduction for the overall cohort and for the different glaucoma types.

**Table 2:** IOP reduction at 12-month and 24-month follow-up visits compared to preoperative values

	12 M (range 9-14 M)	24 M (range 21-26 M)
Total (n)	164	88
Mean (mmHg)	−5.1	−5.4
SD	5.2	6.0
Percent reduction	25.5%	26.6%
p-value	< 0.001	< 0.001
POAG (n)	84	46
Mean (mmHg)	−5.1	− 5.4
SD	5.4	6.1
Percent reduction	25.2%	26.6%
p-value	< 0.001	< 0.001
PEX (n)	42	19
Mean (mmHg)	−5.1	−7.2
SD	4.1	6.2
Percent reduction	25.1%	33.2%
p-value	< 0.001	< 0.001
NTG (n)	18	10
Mean (mmHg)	−3.6	−2.4
SD	5.3	4.6
Percent reduction	21.1%	13.9%
p-value	0.010	0.132
OH (n)	20	13
Mean (mmHg)	−6.9	−5.2
SD	6.0	6.3
Percent reduction	32.4%	25.1%
p-value	< 0.001	0.011

IOP = intraocular pressure; M = month; POAG = primary open angle glaucoma; PEX = pseudoexfoliation glaucoma; NTG = normal tension glaucoma; OH = ocular hypertension; SD = standard deviation; *p-values from paired samples t-test vs. preoperative

**Fig. 2:**
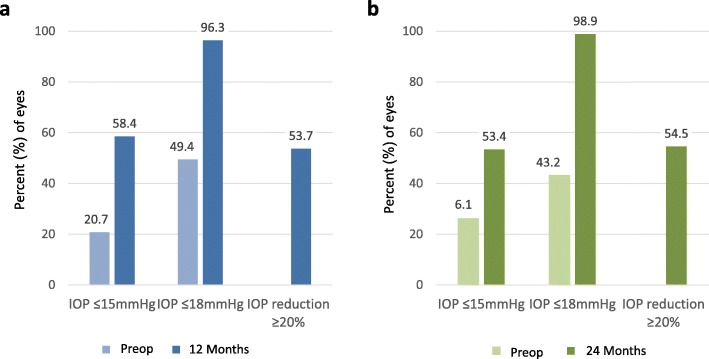
Proportional analysis of eyes with IOP ≤ 15 mmHg, IOP ≤ 18 mmHg, or IOP reduction of 20% or more preoperatively and at (a) 12 months (*n* = 164) and (b) 24 months (*n* = 88) postoperatively. IOP = intraocular pressure; Preop = preoperative

Figure 2 shows the proportions of eyes in the 12-month and 24-month overall cohorts with an IOP ≤ 18 mmHg or an IOP ≤ 15 mmHg through 12 months and 24 months postoperative. At 12 months postoperative, an IOP reduction of ≥20% vs. preoperative was achieved in 88 eyes (53.7%), with 158 eyes (96.3%) achieving an IOP ≤ 18 mmHg and 96 eyes (58.5%) reaching ≤15 mmHg. After 24 months, 48 eyes (54.5%) achieved an IOP reduction of ≥20% vs. preoperative, with 87 eyes (98.9%) achieving an IOP ≤ 18 mmHg and 47 eyes (53.4%) reaching ≤15 mmHg.

### Medication burden evaluation

The mean number of medications in the overall cohort was reduced by 85.0% from 2.0 ± 1.0 preoperatively to 0.3 ± 0.8 in the 12-month consistent cohort (*p* < 0.001), and by 81.0% from 2.1 ± 1.1 to 0.4 ± 0.8 in the 24-month consistent cohort (*p* < 0.001). A decrease in medication burden was observed in 150 eyes (91.5%) at 12 months and 79 eyes (89.8%) at 24 months. Compared to 1.8% of eyes (3 of 164) in the 12-month cohort and 1.1% of eyes (1 of 88) in the 24-month cohort at baseline, 81.1% of eyes (133 of 164) and 72.7% of eyes (64 of 88) were medication-free at 12 months and 24 months, respectively (*p* < 0.001 at both time points). Postoperatively, there were 5 (3.0%) and 2 (2.3%) eyes on ≥ 3 medications at 12 months and 24 months, respectively, compared to 53 eyes (32.4%) and 31 eyes (35.2%) in the 12-month and 24-month cohorts preoperatively (*p* < 0.001 at both time points).

**Fig. 3:**
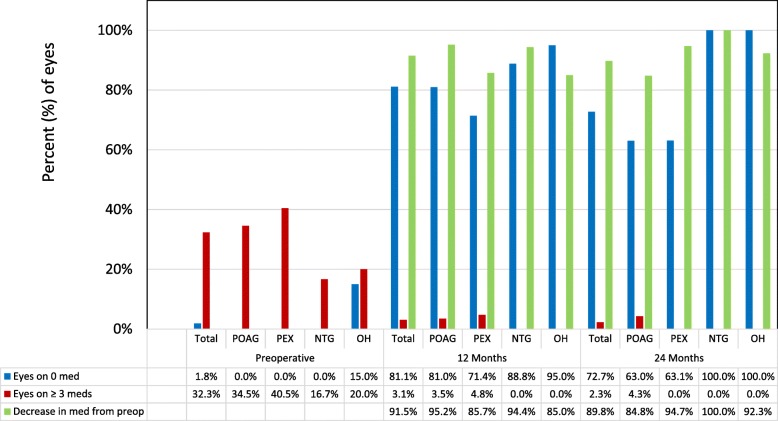
Proportion of glaucoma medications preoperatively (*n* = 164) as well as at 12 (*n* = 164) and 24 months (*n* = 88) postoperatively for the overall group and the different glaucoma types. POAG = primary open angle glaucoma; PEX = pseudoexfoliation glaucoma; NTG = normal tension glaucoma; OH = ocular hypertension; med = medication; Preop = preoperative

A similar analysis was conducted for the glaucoma sub-types. The proportion of medication-free eyes increased significantly from baseline to 12 months and 24 months in all groups of glaucoma types (p < 0.001 at both time points). A significant reduction in the proportion of eyes with ≥ 3 medications could be observed in the POAG and PEX groups at 12 months and 24 months (POAG: p < 0.001 at both time points; PEX: *p* < 0.002 and *p* = 0.008, respectively). In the NTG and OH groups, this proportion also decreased both at 12 months and 24 months, but this was not statistically significant (NTG: *p* = 0.235 at both time points; OH: *p* = 0.114 at both time points) (Fig. 3). In all glaucoma subtypes, medication reduction was achieved in over 84% of eyes at both 12 months and 24 months (Fig. 3).

### Safety

All patients underwent uneventful implantation of 2 iStent inject stents with concomitant phacoemulsification with no intraoperative or postoperative complications. An overall high safety profile was observed with no reports of peripheral anterior synechiae (PAS) or other inflammatory conditions, hypotony, hyphema, and choroidal hemorrhage or effusion. There were no cases of stent extrusion or malpositioning of clinical relevance, and no signs of endothelial damage or decompensation. Optic nerve health was assessed to be stable with no significant change in cup-to-disc (C:D) ratio. Over the 24-month follow-up period, one eye underwent secondary glaucoma intervention (trabeculectomy).

Mean corrected distance visual acuity (CDVA) improved from 0.43 ± 0.14 decimal preoperatively to 0.84 ± 0.19 and 0.79 ± 0.20 decimal postoperatively at 12 months and 24 months, respectively (*p* < 0.001 at both time points). A total of 156 eyes (95.1%) and 84 (95.5%) reached a CDVA of 0.5 decimal or better at 12 months and 24 months, respectively. There were 4 eyes with postoperative CDVA worse than 0.5 at 24 months. At 24 months, none of the eyes decreased the CDVA after iStent implantation, CDVA remained unchanged in 1 eye and improved slightly in the other 3 eyes. None of these changes at any of the time points was statistically significant.

## Discussion

The aim of this retrospective evaluation of iStent inject implanted in combination with cataract surgery was to provide a large dataset of eyes from a single-surgeon case series with a postoperative follow-up of up to 24 months in a routine clinical setting. In contrast to a clinical study with defined inclusion and exclusion criteria, this case series reflects the real life situation in MIGS with trabecular micro-bypass devices. In addition, this study was not limited to patients with primary open angle glaucoma, but also included eyes with other glaucoma types.

With the implantation of iStent inject in 164 eyes with POAG (predominant diagnosis), PEX, NTG and OH, clinically and statistically significant reduction of IOP and medication usage was demonstrated. In the overall cohort, IOP reduction was observed to be greater than 20% representing ~ 5 mmHg reduction out to 24 months. Landmark glaucoma studies have established that every 1 mmHg IOP reduction produces a 10% or 19% lower risk of glaucoma progression [43, 44]. Mean number of medications used was reduced by ~ 80% from baseline and sustained to 24 months. By 24 months postoperatively, the vast majority of eyes (89.8%) of eyes reduced their medication burden compared to baseline, significantly more eyes were medication-free with 72.7% medication-free compared to 1.1% preoperatively, and significantly fewer eyes were on 3 or more medications. Reducing medication burden in any capacity has substantial value given the recognized downsides of medications such as side effects including ocular surface damage, poor adherence, and costs. Thus, the fact that 72.7% of eyes in this cohort were medication-free at 24 months is particularly noteworthy.

The overall benefits of surgery may be interpreted in the context of glaucoma subtype. Within each glaucoma subtype, benefits in IOP or medications could figure more or less prominently. For example, the postoperative IOP reduction in NTG eyes was not as significant as that of the other glaucoma subtypes in this study. This is not surprising given that NTG eyes had a lower baseline IOP than the other subgroups, and lower baseline IOPs are known to result in less postoperative IOP reduction. Nevertheless, NTG eyes had some of the most dramatic reductions in medication burden in the cohort, and all 10 eyes with NTG were medication-free at 24 months after surgery.

The IOP and medication reductions and favorable safety observed in this study are consistent with existing literature on iStent inject, which includes studies of standalone and cataract-combination procedures, different subtypes and severities of glaucoma, and different study designs [30–42]. A large multicenter pooled analysis showed 12-month outcomes of iStent inject implantation with cataract surgery in various types of glaucoma as well as ocular hypertension. IOP reduced by 23.2% to 14.04 mmHg and medications decreased by 71.5% to 0.47 mmHg [32]. In three independent single-surgeon studies with different baseline patient populations, significant IOP and medication reductions were demonstrated following iStent inject with cataract surgery. An Australian case series showed 29.4% IOP reduction and 94.7% medication reduction [41]; a Brazilian case series showed 19.1% IOP reduction and 94.1% medication reduction [33]; and a Canadian study showed 17.8% IOP reduction and 56% medication reduction [42]. In a 3-year prospective case series of patients undergoing iStent inject implantation with cataract surgery in Germany, IOP reduced by 37% (from 22.6 mmHg to 14.3 mmHg) and medications reduced by 68% (a 1.7-medication reduction) [39], with similarly favorable IOP and medication outcomes achieved in POAG and PXG subgroups. Notably, the patient population had a relatively greater disease burden than many other iStent inject studies, with 56% of eyes on 3–4 preoperative medications and 32.1% of eyes with prior glaucoma surgery. The vast majority of eyes in this more advanced population were able to avoid secondary glaucoma surgery, indicating that iStent inject could be a viable treatment option even in eyes with more advanced glaucoma. The same author also published 3-year outcomes of patients undergoing standalone iStent inject implantation and showed IOP reduction of 42% and medication reduction of 87.9% [40].

Alongside these real-world datasets, several prospective controlled trials have evaluated iStent inject. A multicenter European trial showed a 39.7% mean IOP reduction to 14.7 mmHg, with nearly 87% of subjects reducing their medication regimen and over 71% of eyes eliminating ≥2 medications versus preoperative [35]. A prospective randomized controlled study compared iStent inject implantation vs. two topical medications in eyes with open-angle glaucoma (OAG) on one preoperative medication [34]. In the study, standalone iStent inject implantation resulted in a 48% decrease in mean medication-free IOP versus unmedicated baseline IOP, with 94.7% of eyes achieving the efficacy endpoint of ≥ 20% IOP reduction without medication versus baseline. A third study of standalone iStent inject implantation in eyes with OAG on one preoperative medication demonstrated 18-month IOP reduction of 41% and elimination of topical medication in over 98% of eyes [38]. A fourth prospective study evaluated standalone iStent inject implantation combined with topical postoperative prostaglandin in eyes with OAG on two preoperative medications [37]. The study showed 18-month IOP reduction of 37% as well as elimination of one medication in all eyes.

Although the current report pertains to the second-generation iStent inject trabecular micro-bypass, it is instructive to view results alongside long-term data of its first-generation predecessor, the iStent trabecular micro-bypass. Since iStent was the first U.S. FDA-approved MIGS device, it is the only device that currently has safety and efficacy data up till 5 years postoperative. A recent real-world analysis of glaucoma patients within my clinical population showed 5-year outcomes following implantation of one iStent with cataract surgery. Favorable safety and effectiveness were sustained throughout follow-up, including 5-year IOP reductions of 38% and medication reductions of 75% [20]. Another study prospectively randomized newly-diagnosed, treatment-naive patients to either standalone implantation of two iStents or topical prostaglandin [26]. At 5 years postoperative, the 2-iStent group achieved 35.3% IOP reduction along with excellent safety; these outcomes were comparable to the medication group, and yet 83% of iStent patients were medication-free through 5 years.

The rationale behind iStent inject’s dual-stent design lies in preclinical and clinical research. Within a human anterior segment model, IOP reduced from 21.4 to 12.4 mmHg after implanting one iStent (*p* < 0.001), while eyes receiving more than one stent reached a final IOP of 11.9 mmHg [45]. Using a similar model, Bahler et al. showed that one iStent inject stent increased aqueous outflow facility from 0.16 to 0.38 μL/min/mmHg (*p* < 0.03, *n* = 7), with IOP decreasing from 16.7 to 8.6 mmHg, while addition of a second iStent inject stent further improved outflow facility to 0.78 μL/min/mmHg (*n* = 2) [46]. Within a whole eye perfusion model, IOP decreased by 6.0 mmHg from baseline with a single iStent and decreased by an additional 2.9 mmHg with a second iStent, for a total IOP reduction of 8.9 mmHg from baseline [47]. Echoing these preclinical findings, a prospective clinical trial compared 3.5-year results of one, two, or three iStents implanted in a sole procedure [30], while two other studies analyzed two or three iStents with concomitant cataract surgery [48, 49]. These three studies confirmed that while the first stent produces the majority of IOP reduction, additional stents enable further reduction. This is conceivably due to accessing more areas of distal outflow and increasing the chance of implantation into patent and functional areas of Schlemm’s canal.

The iStent inject’s multidirectional design is also supported by research in computational fluid dynamics (CFD), the gold standard technique to analyze microtubule size and flow. CFD studies showed that the flow resistance of each iStent inject stent is negligible, allowing for unhindered IOP reduction [47]. The CFD models estimated that a single stent (either iStent or iStent inject) was completely capable of conducting all 2.5 μL/min of aqueous humor production [47]. Huang et al. confirmed these findings in an aqueous angiography study of patients undergoing iStent inject implantation with cataract surgery, showing that aqueous outflow patterns improved and formerly dormant outflow areas were reactivated, thereby increasing the span of collector channels for outflow [50].

Given the permanent nature of glaucoma and the lengthening of lifespans worldwide, the safety profile of a given glaucoma surgical intervention is critical. Safety was excellent in this study, with no intraoperative nor stent-related complications, and with few mild postoperative adverse events over the course of follow-up. There were no complications such as those seen with conventional filtering surgeries or some other MIGS devices (e.g., endophthalmitis, hypotony, choroidal detachment or effusion, significant hyphema, device dislocation) [6–8, 51–54]. There also were no cases of inflammation (e.g., uveitis, iritis) or PAS, which have emerged as a concern with some MIGS devices, particularly those containing nickel [55–57]. Nickel’s safety risks have led to heavy regulations in Europe, [58–63], and the long-term safety of nickel-containing intraocular implants is still unknown, warranting caution given that approximately 8–19% of the population has a nickel allergy [59]. Furthermore, the findings in our study showed improved visual acuity consistent with expectations for cataract surgery, indicating that stent implantation did not detract from the visual benefits of cataract surgery. Structural function was assessed by C:D ratio and demonstrated stable optic nerve appearance out to 24 months.

This evaluation has some limitations. There was no control group and no washout period prior to the surgery. However, as mentioned before, this setting reflects the real-world situation in glaucoma surgery. The mean IOP reduction at 12 and 24 months postoperatively is compelling and supports the MIGS procedure with the iStent inject.

The results of this 24-month evaluation demonstrate it is possible to reduce the medication burden while maintaining the target IOP in patients with limited medication adherence, medication tolerance and/or dexterity. Importantly, all but one eye in this sample avoided secondary glaucoma surgery through 24 months postoperative. These data suggest that by incorporating MIGS with iStent inject into the glaucoma treatment algorithm, surgeons may be able to postpone more invasive glaucoma surgery, with the advantage of MIGS being less traumatic, having an excellent safety profile, and leaving open all options of further glaucoma treatment.

## Conclusions

The safety and effectiveness results of this evaluation complement the growing body of evidence supporting the use of the second-generation trabecular micro-bypass stent iStent inject as a safe and effective treatment option for patients with mild to moderate open-angle glaucoma.

## Data Availability

The datasets generated and/or analyzed during the current study are not publicly available due to confidentiality of clinical records but are available from the corresponding author on reasonable request.

## Abbreviations

C:D: cup-to-disc
CDVA: corrected distance visual acuity
CFD: computational fluid dynamics
FDA: Food and Drug Administration
IOP: intraocular pressure
MIGS: micro-invasive glaucoma surgery
NTG: normal-tension glaucoma
OAG: open-angle glaucoma
OH: ocular hypertension
PAS: peripheral anterior synechiae
PEX: pseudoexfoliation glaucoma
POAG: primary open-angle glaucoma
VF: visual field
